# Effects of Androgen Receptor and Androgen on Gene Expression in Prostate Stromal Fibroblasts and Paracrine Signaling to Prostate Cancer Cells

**DOI:** 10.1371/journal.pone.0016027

**Published:** 2011-01-18

**Authors:** Matthew J. Tanner, R. Charles Welliver, Mengqian Chen, Michael Shtutman, Alejandro Godoy, Gary Smith, Badar M. Mian, Ralph Buttyan

**Affiliations:** 1 Ordway Research Institute, Albany, New York, United States of America; 2 Division of Urology, Department of Surgery, Albany Medical College, Albany, New York, United States of America; 3 Stratton Veterans Affairs Medical Center, Albany, New York, United States of America; 4 Department of Urology, Roswell Park Cancer Institute, Buffalo, New York, United States of America; Weill Cornell Medical College of Cornell University, United States of America

## Abstract

The androgen receptor (AR) is expressed in a subset of prostate stromal cells and functional stromal cell AR is required for normal prostate developmental and influences the growth of prostate tumors. Although we are broadly aware of the specifics of the genomic actions of AR in prostate cancer cells, relatively little is known regarding the gene targets of functional AR in prostate stromal cells. Here, we describe a novel human prostate stromal cell model that enabled us to study the effects of AR on gene expression in these cells. The model involves a genetically manipulated variant of immortalized human WPMY-1 prostate stromal cells that overexpresses wildtype AR (WPMY-AR) at a level comparable to LNCaP cells and is responsive to dihydrotestosterone (DHT) stimulation. Use of WPMY-AR cells for gene expression profiling showed that the presence of AR, even in the absence of DHT, significantly altered the gene expression pattern of the cells compared to control (WPMY-Vec) cells. Treatment of WPMY-AR cells, but not WPMY-Vec control cells, with DHT resulted in further changes that affected the expression of 141 genes by 2-fold or greater compared to vehicle treated WPMY-AR cells. Remarkably, DHT significantly downregulated more genes than were upregulated but many of these changes reversed the initial effects of AR overexpression alone on individual genes. The genes most highly effected by DHT treatment were categorized based upon their role in cancer pathways or in cell signaling pathways (transforming growth factor-β, Wnt, Hedgehog and MAP Kinase) thought to be involved in stromal-epithelial crosstalk during prostate or prostate cancer development. DHT treatment of WPMY-AR cells was also sufficient to alter their paracrine potential for prostate cancer cells as conditioned medium from DHT-treated WPMY-AR significantly increased growth of LNCaP cells compared to DHT-treated WPMY-Vec cell conditioned medium.

## Introduction

The prostate gland requires androgenic steroids for development, adult maintenance and function. Males with inactivating mutations in key genes required for androgen metabolism develop only a rudimentary prostate gland [1] and males with inactivating mutations in the androgen receptor (AR) gene, that mediates the effects of androgens, do not develop prostates [2]. Androgens and AR action also play an important role in prostate carcinogenesis. Drugs that inhibit androgen biosynthesis have chemopreventative effects that significantly reduce the risk for developing prostate cancer in men [3] and androgen ablation therapies provide the most clinically useful means for palliative disease control when prostate cancer is detected in the advanced stage [4]. These clinical facts identify the relevance of androgen signaling for prostate biology and carcinogenesis and drive research efforts to characterize the consequences of androgen signaling in prostate cells.

Since the AR protein is an extended member of the nuclear transcription factor that conditionally regulates the expression of genes [5], it is reasonable to expect that the availability of a comprehensive catalogue of androgen regulated genes in prostate cells could significantly contribute to our knowledge of androgen action in the prostate. To this end, the use of contemporary mass gene expression profiling technology, especially involving gene microarrays on Chips, has already greatly expanded the list of known androgen regulated genes in prostate cancer cells [6]–[9]. Studies using this approach have supported the eventual identification of novel genetic anomalies (ETS gene rearrangements) [10]–[12] and have helped to identify abnormally active signaling pathways in prostate cancer cells [13], [14] that have translational potential for improving prostate cancer diagnostic or treatment strategies. This type of technology, however, has not yet been used to characterize androgen/AR effects on gene expression in prostate stromal cells, despite the extensive evidence that cells from the prostate stroma actively participate in the processes through which androgens regulate normal or malignant prostate development [15]–[19]. The principal reason for this deficit is the lack of suitable cultured human prostate stromal cell models that robustly express the AR protein and are demonstrably responsive to the presence of androgens as indicated by changes in gene expression when cultured in an androgen containing medium. Here, we describe our experience in testing some available (benign) human prostate stromal cell models for their responsiveness to androgens *in vitro* and in developing a specific androgen-responsive human prostate stromal cell model (WPMY-AR cells) that was profiled for AR- and androgen-induced changes in gene expression using human gene Chip microarrays. Furthermore, we used this model cell system to test the idea that androgens alter the paracrine signaling environment of a prostate tumor by affecting the output of secreted factors from prostate stromal fibroblasts.

## Results

### Androgen receptor expression and activity in cultured human prostate stromal cells

**Figure 1:**
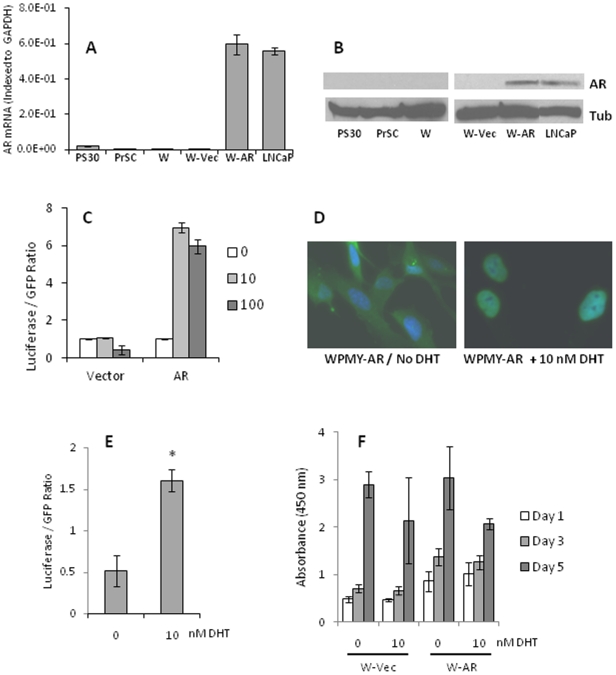
Androgen receptor expression and activity in prostate stromal cell lines. (A) AR mRNA levels in PS30, primary prostate stromal (PrSC), WPMY-1 (W), WPMY-Vec (W-Vec), WPMY-AR (W-AR) or LNCaP cells detected by real-time qPCR of RNAs extracted from the cells. Expression levels are indexed to the expression of GAPDH in each cell line. (B) AR protein (upper lanes) in PS30, PrSC, W, W-Vec, W-AR or LNCaP cells detected by Western blot. The blot was re-probed for GAPDH protein (lower lanes) as a control. (C) Luciferase reporter expression in WPMY-1 cells co-transfected with the ARE-luc reporter vector and a control (empty) vector (Vector) or the pLenti6.2-hAR vector (AR). Luciferase levels are normalized for GFP fluorescence in the same extract as the transfection control marker. (D) Immunofluorescent staining for AR in W-AR cells grown for 72 hrs in the absence (left) or presence (right) of 10 nM DHT. Cells were co-stained with DAPI to identify nuclei. (E) Luciferase activity in W-AR cells transfected with ARE-Luc and GFP in the absence or presence of 10 nM DHT. Luciferase activity was normalized by comparison to GFP levels in the same extract. (F). Growth of W-Vec or W-AR cells in the absence or presence of 10 nM DHT as measured by the WST-1 assay.

Two available immortalized human prostate stromal cell lines, PS-30 and WPMY-1, and non-immortalized primary human prostate stromal cell myofibroblasts were evaluated for AR expression and responsiveness to androgens. None of these cells require androgen for *in vitro* growth, however, WPMY-1 cells were previously reported to grow slightly faster in the presence of synthetic androgen, R1881 [20]. AR expression was assessed in these cells by quantitative RT-PCR (qPCR) and Western blot procedures and was compared to cultured primary human prostate stromal fibroblasts (PrSC) and to LNCaP prostate cancer cells that are models for AR action in prostate cancer (Figure 1A). Of the surveyed cells, LNCaP cells expressed the highest levels of AR mRNA. AR mRNA was expressed at only 3.4% of this level in PS30 cells, 1.1% in PrSC and at slightly over 0.1% of this level in WPMY-1 cells. This pattern was consistent with our Western blot data where we were unable to detect a band corresponding to AR in extracts of either of the immortalized cells or in PrSC though it was readily detected in the extract from LNCaP cells (Fig. 1B). Likewise, when parental WPMY-1 cells were transfected with an androgen responsive reporter vector, they showed no evidence of increased expression of the reporter (luciferase) in response to increasing amounts of DHT (Fig. 1C). However, when WPMY-1 cells were co-transfected with the androgen reporter along with an AR expression vector, the expression of the reporter was significantly increased by the presence of DHT (Fig. 1C). In summary, the low endogenous AR expression in these human prostate stromal cell lines and their unresponsiveness to androgen stimulation suggests that they are poor models for the study of androgen action in stromal cells, but exogenous expression of AR, at least in the WPMY-1 cells, conferred upon these cells an androgen-responsive phenotype that could be more conducive to the study of androgen action.

In order to make WPMY-1 cells more amenable for the study of androgen effects on gene expression, we transduced the cells with human wildtype AR expression lentivirus and then used antibiotic selection to obtain a stable population of AR overexpressing WPMY-1 cells (WPMY-AR). Other WPMY-1 cells were transduced with empty lentivirus and selected under the same conditions to obtain a control cell population (WPMY-Vec). WPMY-AR cells express AR mRNA and protein at a level comparable with androgen-sensitive LNCaP prostate cancer cells (Figs. 1A, B). Immunofluorescence staining using anti-AR antibody showed that AR was mostly in the cytoplasm when these cells were grown in the absence of DHT, although there was light nuclear immunofluorescent staining in most cells (Fig. 1D). In contrast, when WPMY-AR cells were grown in DHT-containing medium, AR immunostaining was exclusively nuclear. The AR expressed in the stable WPMY-AR cells was functional for genomic activation of gene expression. When these cells were transfected with the androgen-reporter, luciferase activity was significantly increased by treatment with DHT whereas DHT did not affect luciferase expression in reporter-transfected WPMY-Vec control cells (Fig. 1E). Otherwise, WPMY-AR cells showed no other overt phenotypic differences when compared to WPMY-Vec control cells; they were indistinguishable by morphology under microscopic observation (not shown) and have similar growth rates in both androgen-free and androgen-containing medium (Fig. 1F).

### Comparative Gene Expression Profiling of Prostate Stromal Cell Variants Grown in the Presence or Absence of DHT

**Figure 2:**
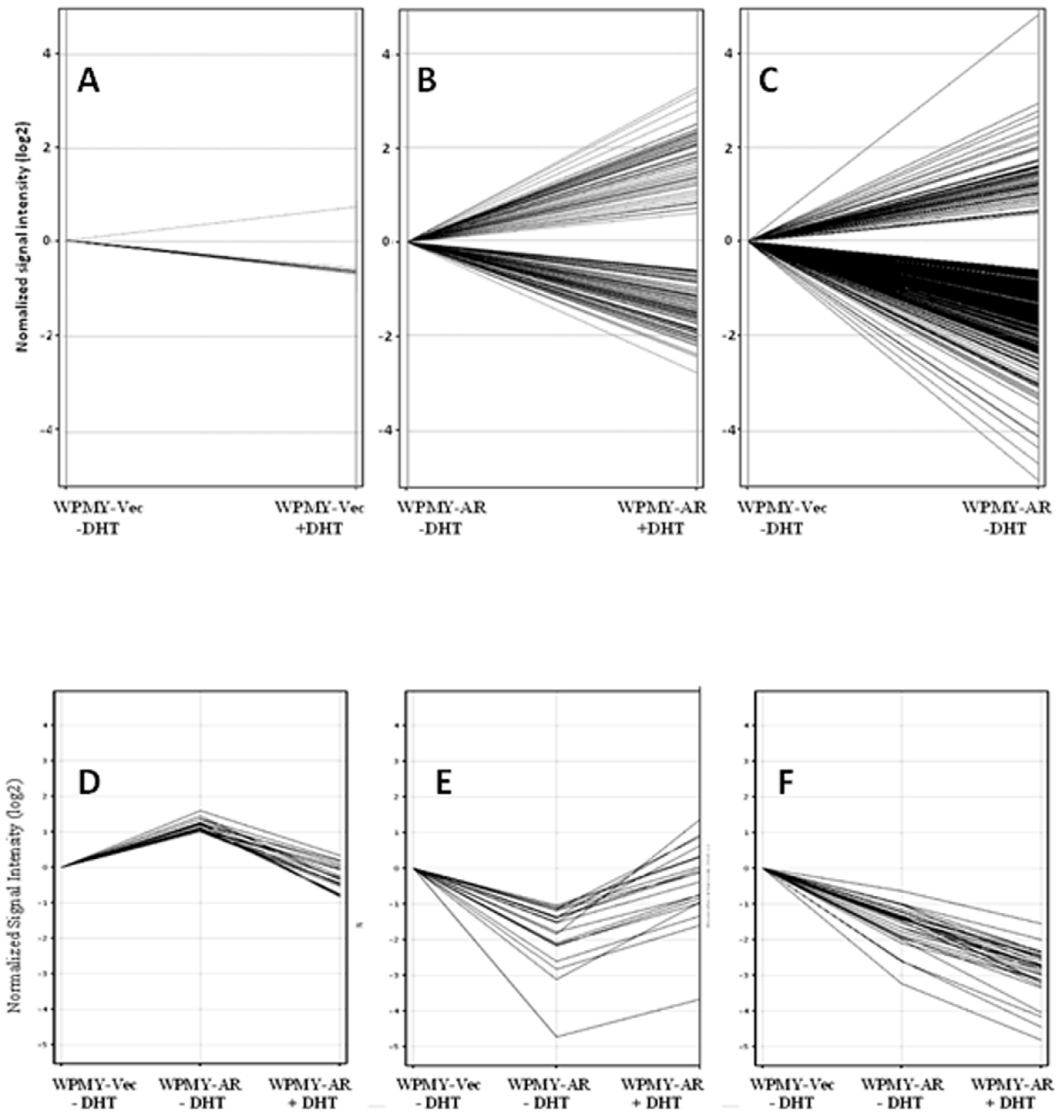
Gene expression changes associated with expression of AR in the absence or presence of DHT in WPMY-1 prostate stromal cells. (A) GeneSpring-generated line plot of significant (P<0.05) gene expression differences greater than 1.5-fold in WPMY-Vec cells treated for 72 hrs with 10 nM DHT. (B) GeneSpring-generated line plot of significant (P<0.05) gene expression differences greater than 1.5-fold in WPMY-AR cells treated for 72 hrs with 10 nM DHT. (C) GeneSpring-generated line plot of significant (P<0.05) gene expression differences greater than 1.5-fold between WPMY-Vec and WPMY-AR cells grown without DHT. (D) GeneSpring-generated line plot showing effect of DHT treatment on genes that were differentially upregulated by 2-fold or greater by AR expression alone (no DHT). (E) GeneSpring generated line plot showing effect of DHT treatment on genes that were differentially down-regulated by 2-fold or greater by AR expression alone (no DHT) and were subsequently up-regulated by 2-fold in the presence of DHT. (F) GeneSpring generated line plot showing effect of DHT treatment on genes that were differentially down-regulated by 2-fold or greater by AR expression alone (no DHT) and were subsequently further down-regulated by 2-fold or greater in the presence of DHT.

**Figure 3:**
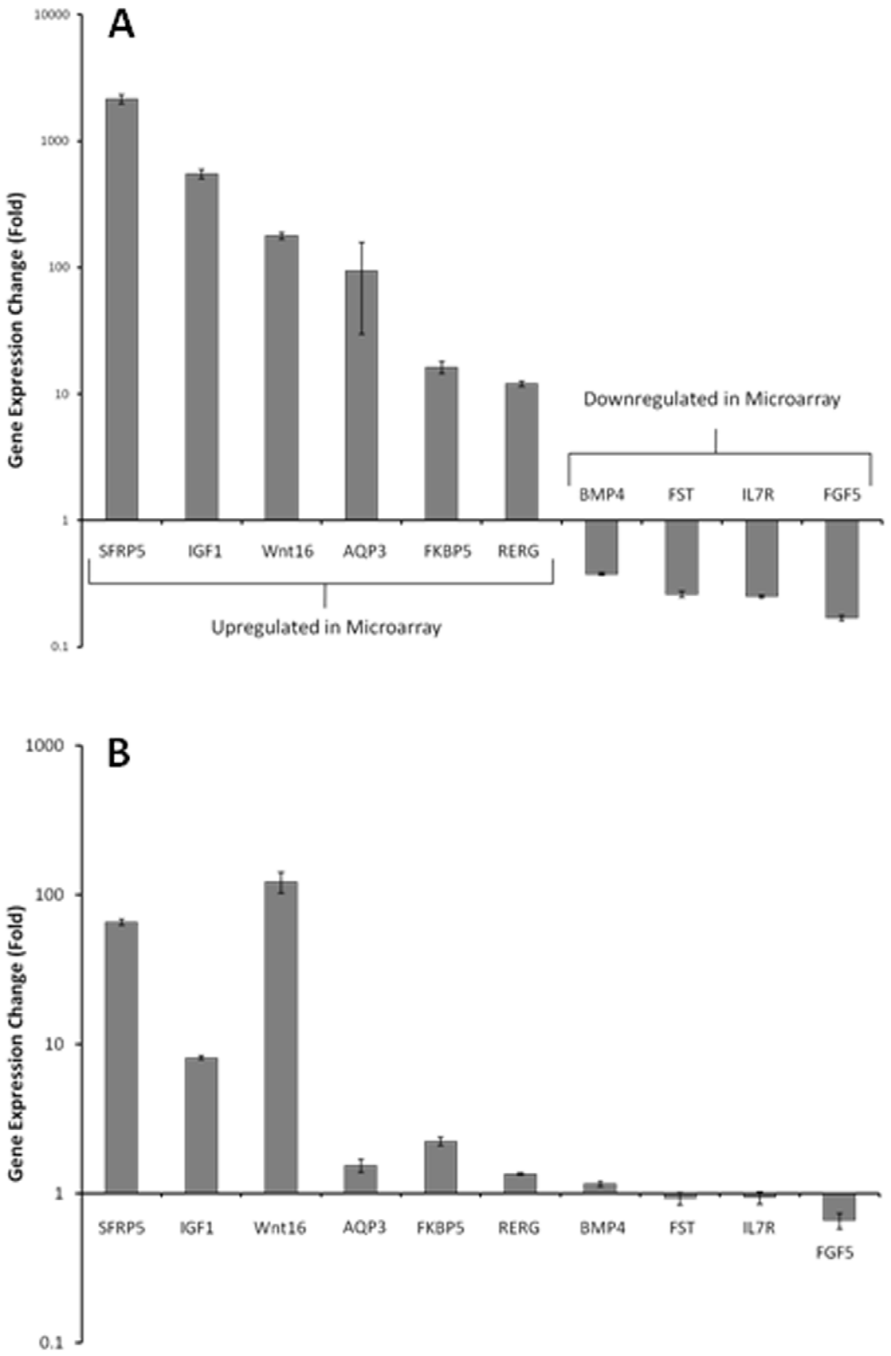
Confirmation of microarray-identified androgen-regulated genes (>2-fold changed) by real-time qPCR measurement. (A). Assessment of individual gene expression changes associated with DHT treatment of WPMY-AR cells by qPCR. Six of the genes in this panel (SFRP-5, IGF-1, Wnt-16, AQP3, FKBP5 and RERG) were identified as DHT-up-regulated genes in the microarray gene expression analysis and four genes (BMP-4, FST, IL7R and FGF5) were identified as DHT-down-regulated genes in the microarray gene expression analysis and these changes were confirmed in the qPCR assay. All changes detected by qPCR were significant changes (P<0.05). (B) Assessment of individual gene expression changes associated with DHT treatment of PS30 cells transiently transfected with pLenti6.2-hAR by qPCR. Measurement of changes in SRBP5, IGF1, Wnt-16, AQP3, FKBP5, RERG and FGF5 were significant (P<0.05) whereas changes in BMP-4, FST and IL7R were not significant (P>0.05).

WPMY-Vec and WPMY1-AR cells were plated in equal numbers in androgen-free medium for attachment then transferred to fresh medium with or without supplemental 10 nM DHT for 72 hrs. RNAs extracted from biological duplicates of these cultures were labeled then profiled on Affymetrix Human Gene ST 1.0 Array Gene Chips. The microarray expression data was analyzed to identify those genes that were differentially expressed between a given cell under differing conditions (−/+ DHT) or between the two cell types (WPMY-Vec vs WPMY-AR) under equivalent conditions. Using a cutoff of 1.5-fold changes in RNA expression, WPMY-Vec control cells had only 8 genes that were differentially expressed in the presence of DHT and the graph showing the range of these changed genes was generated by the GeneSpring program and is shown in Figure 2A. We attempted to confirm differential expression of these 8 genes in WPMY-Vec DHT-treated/-untreated cells using real-time qPCR to assess expression of each gene on a fresh set of biological duplicate samples but the outcomes of this analysis showed no significant differences in expression for any of them using this method (not shown). Comparison of the gene expression profiles of DHT-treated/-untreated WPMY-AR cells, however, did show much more striking and robust changes in gene expression associated with DHT treatment. DHT affected the expression of 172 individual genes by 1.5-fold or greater (Fig. 2B). However, the majority of these changes (141 or 81.9%) were at the level of 2-fold or greater. In this latter category, more genes were downregulated by DHT (85 genes) than were upregulated (56 genes). The genes that were changed by 2-fold or greater are identified in Table S2 and Table S3. We then chose 10 different genes from these lists, including 6 upregulated and 4 downregulated genes, for further validation by real-time qPCR on a fresh set of RNAs extracted from biological duplicate samples. Each of these selected genes was confirmed to be significantly up- or down-regulated by the presence of DHT in the same manner as the results of the microarray expression analysis (Fig. 3A). Finally, the list of genes (up- and down-regulated) that were changed by 2-fold or greater in the presence of DHT was functionally assessed using the *Pathway Express* software program (http://vortex.cs.wayne.edu/projects.htm) [21] that assigns genes into specific KEGG functional pathways and then the different KEGG pathways associated with these genes were quantitatively prioritized by either of two different parameters: 1) the number of input genes that are assigned to a specific KEGG pathway; or 2) the percent of individual KEGG pathway genes that were present in the input gene set (Table 1). The top 10 KEGG pathway rankings using the two different parameters shared the categories, Pathways in Cancer, Cytokine-Cytokine Receptor Interaction, TGF-β Pathway, Wnt Pathway, and Hedgehog Signaling Pathway but other prominent cell signaling pathways were represented in one ranking or the other.

**Table 1:** Hierarchy of KEGG pathway assignments of genes significantly changed by 2-fold or greater in WPMY-AR cells treated with DHT.

Top KEGG Pathway Ranking Based Upon the Number of Input Genes in Pathway	# Input Genes In Pathway	Top KEGG Pathway Ranking Based Upon the Percent Of Pathway Genes in Input	% Pathway Genes in Input
Pathways in Cancer	8	TGF-β Pathway	5.747
Cytokine-Cytokine Receptor Interaction	6	Hedgehog Signaling Pathway	3.509
TGF-β Pathway	5	Hematopoietic Pathway	3.448
MAPK Signaling Pathway	5	Cell Adhesion Molecules	2.985
Regulation of Actin Cytoskeleton	4	Wnt Signaling Pathway	2.632
Wnt Signaling Pathway	4	Focal Adhesion	2.463
Neuroactive-Ligand Receptor Pathway	4	Pathways in Cancer	2.424
Hematopoietic Pathway	3	ECM-Receptor Interaction	2.381
Insulin Signaling Pathway	2	Cytokine-Cytokine Receptor Interaction	2.281
Hedgehog Signaling Pathway	2	Type II Diabetes Mellitus	2.222

Genes listed in Table S1 and Table S2 were input into the gene classification alogorithm found at the Pathway Express site (http://vortex.cs.wayne.edu/projects.htm) to rank the KEGG pathway assignments based upon the numbers of input genes in any given pathway or based upon the percentage of input genes in any given pathway and the rankings were concordant.

To better determine whether these gene changes associated with DHT treatment were specific for the WPMY-AR cells or whether they might also occur in other human prostate stromal cells with sufficient AR expression, we transiently transfected PS30 cells with the AR expression vector or an empty control vector then treated these cells without or with 10 nM DHT for 72 hrs. RNAs extracted from these cells were tested by real-time qPCR analysis for expression of AR and for expression of the same 10 genes that were selectively analyzed in WPMY-AR cells. The outcomes showed that AR was expressed 923-fold more in AR-transfected than in control-transfected PS30 cells. As is shown in Figure 3B, expression of 6 of the other 10 genes were changed in the same manner as for the WPMY-AR cells treated with DHT, whereas 4 of the 10 genes were not significantly changed between untreated- or DHT-treated cells. Finally, the primary human prostate cell fibroblasts were also cultured in medium with or without DHT for 72 hrs and RNAs were extracted for real-time qPCR analysis. The cDNAs from these cells were then assayed for DHT effects on expression of 5 different genes from our panel. The outcomes showed that SFRP5 and IGF1 were upregulated by 1.67- to 1.73- fold by DHT (p<0.05) and FGF5 was downregulated by 1.5-fold (p<0.05) compared to no-DHT controls whereas expression of FST and Wnt16 was not significantly changed by DHT treatment of these cells.

### Gene Expression Changes Associated with Overexpression of AR in WPMY-1 Cells

To determine whether AR expression (in the absence of ligand) affected gene expression in the WPMY cells, we also compared the gene expression profiles between WPMY-Vec and WPMY-AR cells grown without DHT treatment. Remarkably 443 genes were found to be differentially expressed between these cells at a level of 1.5-fold or greater (Fig. 2C) and 374 of these genes are differentially expressed by 2-fold or greater between these cells. In this latter subset, 55 genes were selectively upregulated and 319 genes were selectively downregulated in the AR-expressing cells. It was of further interest to determine how these two categories of genes were subsequently affected by DHT treatment. First, we selected those genes (55) that were upregulated by overexpression of AR (at least 2-fold) in the absence of DHT. Sixty percent of these genes (33 genes) were subsequently downregulated (by 2-fold or greater) again in the presence of DHT (Fig. 2D) whereas the other 40% were either unchanged or changed less than 2-fold by DHT and, therefore, excluded from our analysis. For those 319 genes that were downregulated by 2-fold or greater by AR overexpression alone, 21 genes (6.58%) were subsequently upregulated by 2-fold or greater by the addition of DHT (Figure 2E) whereas 21 genes (6.58%) were further downregulated by 2-fold or greater by the addition of DHT (Figure 2F). The remaining genes in this category (277 or 86.8%) were either unchanged by addition of DHT or were changed less than 2-fold and excluded from our analysis. No genes were upregulated by AR expression then further upregulated by DHT even in those that were affected by DHT<2- to 1.5-fold. In summary, AR overexpression alone in the absence of ligand can induce but mainly repress expression genes in WPMY-1 cells, but these effects were sometimes reversed in the presence of ligand. However, some gene expression changes induced by AR overexpression (gene downregulations) were further augmented by the treatment with the androgen ligand in these cells.

### Direct or Indirect Regulation of Genes by DHT

**Figure 4:**
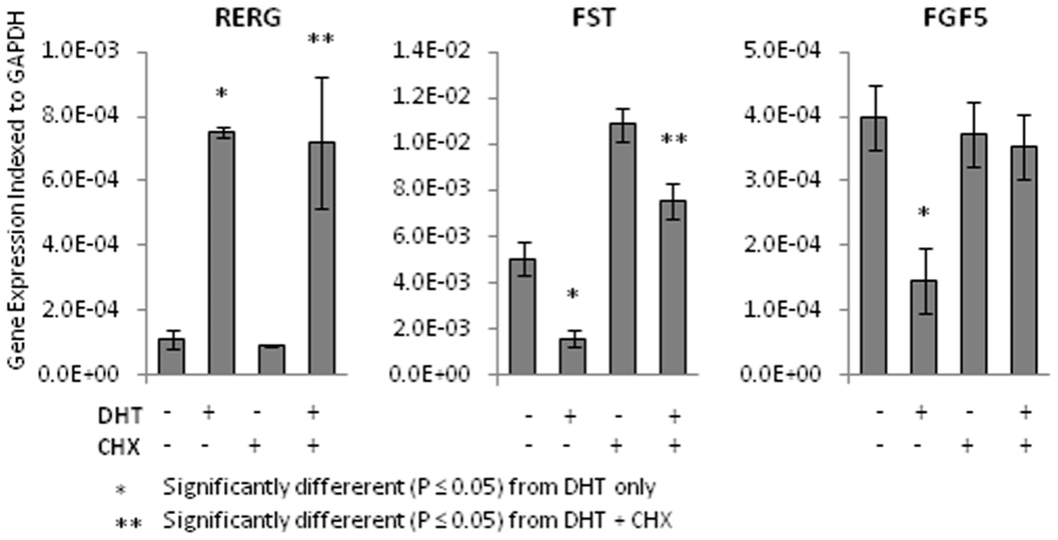
Effects of cycloheximide on gene expression changes in WPMY-AR cells induced by DHT treatment. WPMY-AR cells were pre-treated then treated with cycloheximide in the absence or presence of 10 nM DHT for 24 hrs. RNAs were analyzed by qPCR for the expression of RERG, FST or FGF5, as indicated and expression levels were normalized to GAPDH expression levels.

We described here altered patterns of gene expression in prostate stromal cells induced by AR overexpression, with or without ligand, that were based upon measurements of mRNA levels. We sought further to evaluate a small subset of these DHT-regulated genes to determine whether the effects of DHT required intermediary protein synthesis. To this end, trypsinized WPMY-AR cells were allowed to attach overnight and then briefly treated (30 min) with high dose cycloheximide (40 µgs/ml) to block protein synthesis and thereafter switched to medium with or without DHT (10 nM) in the presence of lower dose cycloheximide (10 µgs/ml) for 24 hrs. Control cells were treated similarly except that no cyclohexmide was included at any time. RNAs extracted from these cells were then assessed for expression of select DHT-upregulated (RERG, Wnt16 and SFRP5) or DHT-down-regulated (FST, FGF5 and BMP4) genes. Our results (Figure 4) showed that the DHT effect on expression changes for four of these genes (RERG, WNT16, SFRP5, and FST) were not changed by cycloheximide treatment, whereas the DHT effects on BMP4 and FGF5 expressions were blocked by cycloheximide.

### Effects of DHT-Stimulated WPMY1-AR Conditioned Media on LNCaP Cell Growth

**Figure 5:**
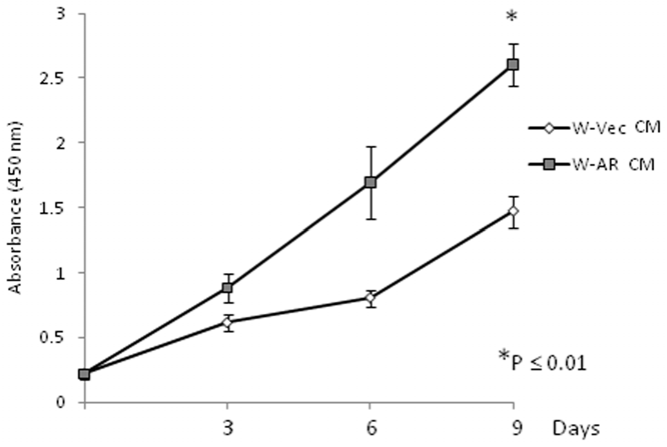
Growth curves of LNCaP cells in the presence of DHT-treated WPMY-AR conditioned medium (W-AR) or DHT-treated WPMY-Vec conditioned medium (W-Vec). Relative cell numbers at different days were estimated by WST-1 assay. Use of conditioned medium from DHT-treated WPMY-AR cells significantly stimulated growth (P<0.01, two way ANOVA) of LNCaP cells compared to conditioned medium from DHT-treated WPMY-Vec cells. Slopes of the two growth curves were also significantly different (P = 0.0181, Linear Regression Analysis).

Finally, we sought to test whether DHT action in the WPMY-AR model cells might affect the production of secreted factors from these cells that influence prostate cancer cell growth. Three day conditioned medium from 10 nM DHT-treated WPMY-Vec or WPMY-AR cells was diluted 1∶1 with fresh medium (with 10 nM DHT) and was then added to fresh LNCaP cells monolayers and the cells were followed for 9 days with medium replacement every 3 days. Growth over this period was measured using the WST-1 assay and results are shown in Figure 5. Treatment with the conditioned medium from the DHT-treated WPMY-AR cells was found to be significantly more growth-stimulatory for LNCaP compared to treatment with conditioned medium from DHT-treated WPMY-Vec cells.

## Discussion

Like other tissues, the prostate is made up of an admixture of disparate cell types that are broadly segregated into an epithelial or a stromal compartment based upon their localization with regards to the basement membrane. Prostate cancer cells that are derived from the prostate epithelium have historically provided the models to study how androgen action affects prostate cell gene expression. However, several cell types within the prostate stroma are also known to express AR *in vivo* [22]–[24] and to contribute to the process(es) through which androgens regulate prostate development and disease yet we know very little regarding the effects of androgen on gene expression in these types of cells. Efforts to this end are hindered by the lack of suitable cultured stromal cell models, especially ones that express AR at sufficient levels to allow the use of contemporary mass gene expression profiling techniques. Here, we attempted to characterize AR expression and androgen signaling activity in two available immortalized prostate stromal cell lines, PS30 and WPMY-1, that were previously reported to express AR [20], [25] to assess whether they might provide models to study androgen regulated gene expression. These cells are both classified as myofibroblasts based upon their morphology in culture and their co-expression of vimentin and smooth muscle actin. We found that both types of cells express extremely low levels of AR mRNA and protein and neither cell type responded to DHT treatment after transfection with an androgen-responsive luciferase reporter vector so neither is likely a good model for studying androgen regulated gene expression. However, when the androgen reporter vector was co-transfected with a wildtype AR expression vector, WPMY-1 cells were then able to respond to DHT treatment by upregulating androgen-responsive reporter expression. This result showed that WPMY-1 cells might be made amenable for study of androgen regulated gene expression when provided with exogenous AR. Transduction by an antibiotic-selectable AR-expression lentivirus allowed us then to derive a stable cell line, WPMY-AR, that expressed AR mRNA and protein at a level comparable to LNCaP cells that are often used to model a prostate cancer cells' response to androgens. The WPMY-AR cells relocated AR protein to the nucleus in the presence of DHT and appropriately upregulate luciferase expression from an androgen-regulated reporter vector after DHT treatment identifying that they have a functional androgen signaling system that is consistent with their use in gene expression profiling experiments. It was notable that the WPMY-AR cells were morphologically indistinguishable from parental or control transduced (WPMY-Vec) cells and that their relative growth rate (in the presence or absence of androgen) was not significantly affected by AR overexpression, especially since AR is known to affect prostate cancer cell growth, either when it is expressed endogenously or exogenously [26], [27].

The WPMY-Vec and WPMY-AR cells were then profiled for overall gene expression patterns and for changes in these patterns associated with DHT treatment using a gene Chip microarray approach. Our preliminary effort involved a more prolonged treatment with DHT (72 hrs) than is commonly used in studies of prostate cancer cells, but we hoped that this longer treatment period would also allow detection of potential secondary gene expression changes that might be relevant to aspects of cross-talk between prostate stromal and epithelial cells that affect prostate development or prostate cancer cell growth. For the WPMY-Vec control cells, DHT treatment significantly altered the expression of only 8 genes (out of 28,712 gene probe sets on the Chip) and these changes were relatively low, ranging from a 1.62- to 1.74-fold change compared to untreated WPMY-Vec cells. None of these gene changes were subsequently confirmed using a real-time qPCR approach. We feel then, that these minor changes in our control cells (+/− DHT) detected using the gene Chip microarray approach represent the “noise” of the system and that this noise is extremely low and easily filtered using the secondary qPCR approach. In striking contrast, treatment of WPMY-AR cells with DHT altered the expression of 141 different genes by 2-fold or greater. The vast majority of these changes (60.2%) involved gene expression down-regulations associated with DHT treatment. Considering that transcriptionally active (liganded) AR is most often thought of as an inducer of gene expression, this is a remarkably high number of potentially androgen-repressed genes. However, AR/androgen repressed genes have been previously described in prostate cancer cells [8] and one report describing the effects of androgen on gene expression in LNCaP cells did show that androgen treatment suppressed almost as many genes as were induced in these cells [9] so our observation is supported by observations in other prostate cell systems. It was also interesting that a comparison of our DHT-changed stromal cell gene lists with already known androgen-regulated genes assembled at a website resource (http://argdb.fudan.edu.cn/index_info.php, [28] showed that approximately 21% of the genes present on our lists (Tables S1 and S2) were previously described to be “androgen regulated” based on surveyed literature sources mainly involving studies of prostate cancer cells. The presence of this significant percentage of previously described “androgen regulated” genes on our lists supports the idea that we are identifying many genes that may be commonly regulated by liganded AR in many types of prostate cells as well as genes that may be selectively affected by AR/androgens in prostate stromal cells.

With regards to the nature of these DHT-regulated stromal cell genes, there were few, if any genes that are functionally classified as regulators of cell proliferative processes or apoptosis and this is consistent with our observations that androgen treatment did not significantly affect WPMY-AR growth. The assessment of gene function for the up-regulated/down-regulated genes on our lists based upon KEGG pathway designation was remarkable since it identified a predominance of genes that are involved in generic “cancer pathways” that might be relevant to our findings that conditioned medium from DHT-stimulated WPMY-AR cells affected LNCaP cell growth. Likewise, the presence of multiple genes on our list classified as effectors of the cytokine-cytokine receptor signaling, TGF-β, WNT, Hedgehog or MAP Kinase signaling pathways would support previous published studies suggesting that these particular signaling pathways are involved in the cross-talk that occurs between prostate stromal and prostate epithelial/cancer cells in development or disease [29]–[31]. Categorization of genes on the list based upon Gene Ontology (GO) assignments were also done using the DAVID program and the outcome showed similar categories to those assigned under the KEGG Pathway (not shown). Finally, our limited survey for an effect of cycloheximide on DHT-induced gene changes does support the idea that many of the gene changes we observed are primarily associated with AR functional activity. Yet our ability to identify some DHT-affected genes in WPMY-AR cells that were not changed when cycloheximide was included with DHT treatment also shows that there are genes on our lists that are secondarily regulated by some other protein affected by DHT as we suspected. Use of WPMY-AR cells with a shorter period of DHT treatment may help us sort out the primary affected vs the secondary affected genes and we will attempt this in the future.

For validation purposes, we had selected a panel of 10 genes from our lists of DHT-changed genes and all 10 of them were confirmed to be appropriately changed by DHT using an alternate assay (real-time qPCR). We believe that this limited effort helps validate the outcome of the overall gene expression profiling for androgen regulated prostate stromal cell genes, especially when we focus on those genes that were changed by 2-fold or greater. Moreover, 7 of these 10 select genes were similarly changed by DHT when we assessed a different prostate stromal cell line, PS30, that was only transiently transfected with the AR expression vector. Considering that the WPMY-AR cells were more enriched for AR expressing cells by stable antibiotic selection, it is possible that all 10 genes in this panel would be similarly regulated if we had also selected the AR-expressing PS30 population with antibiotic. However, this outcome still indicates that there is effective similarity in gene changes induced by liganded AR in WPMY-1 cells as in the PS30 cells. Finally, 4 of 7 genes from this panel were also shown to be regulated by DHT in a similar manner in cultured primary prostate stromal cells that were not manipulated to overexpress AR. Although these primary prostate fibroblasts express AR mRNA in a similar range to the PS30 and WPMY-1 cells, we were, at least, able to show that these cells had increased nuclear AR immunostaining (Figure S1) when they were cultured in DHT so this supports the idea that endogenous AR in primary prostate stromal cells operates in a similar fashion to exogenous AR in the WPMY-AR cells.

Finally, our results were noteworthy in that they showed a significant effect of AR expression alone (in the absence of ligand) on the gene expression patterns of WPMY-1 cells. In fact, there were more gene changes between control (WPMY-Vec) cells and WPMY-AR cells than were found after DHT treatment of WPMY-AR cells. The gene changes associated with AR overexpression alone were even more highly repressive than after DHT treatment in that 85% of genes changed by AR overexpression alone involved gene down-regulation. This raises some concern since our immunostaining work showed that most of the AR expressed in WPMY-AR was cytoplasmically localized in the absence of DHT. It may be that the unliganded AR has an effect on gene expression in WPMY-AR cells through a non-genomic pathway similar to that described in some types of prostate cancer cells where the receptor interacts with cell membrane complex to effect gene changes [32]. However, since there was, at least, minimal nuclear AR immunostaining in non-treated WPMY-AR cells that was not observed in cells similarly stained with IgG non-immune antibody, it is more likely that some exogenous AR expressed in WPMY-AR was afforded access to the nucleus where it affected gene expression patterns through genomic interactions that were significantly more repressive of gene expression in the absence of ligand. Genomic action of unliganded AR in our model is also supported by the fact that addition of DHT reversed or amplified (by at least 2-fold) the expression of 23.5% of the genes that were changed by AR expression alone.

Finally, we attempted to test whether DHT treatment affects the WPMY-AR cells by altering their output of soluble factors that influence prostate cancer cell growth. We chose LNCaP as the cancer test cell model despite the complication introduced by its own endogenous androgen sensitivity because it best represents the phenotype of the prostate cancer cell found in the natural situation. To address the complication of LNCaP's endogenous androgen sensitivity, our approach involved the use of conditioned medium from WPMY-AR or WPMY-Vec cells, both treated with DHT, that was then supplemented into fresh medium that also contained DHT. Here, the WPMY-AR conditioned medium significantly increased the growth of the LNCaP cells over 9 days, compared to the WPMY-Vec conditioned medium. Consistent with this result, we found that LNCaP cells grown for 7 days in WPMY-AR conditioned medium expressed 2.7-fold less p21 mRNA than cells grown in WPMY-Vec conditioned medium. The outcome of this experiment implies either that androgen action selectively increased the production and release of some factor from WPMY-AR cells that increased LNCaP growth or that it reduced the production of some inhibitory factor (made more abundantly by WPMY-Vec cells) that suppresses LNCaP growth. Regardless of the mechanism, this experimental outcome further supports the idea that androgen action in AR-positive fibroblasts has consequences for prostate cancer growth.

In summary, we believe that our efforts represent a step towards identifying the role of AR/androgens in prostate stromal cell gene expression and prostate biology. We have created a cell model to study androgen action on prostate stromal cell genes and we have shown that this model cell responds to androgen stimulation in some ways that are sometimes similar to prostate cancer cells but mostly differs significantly from prostate cancer cells that are usually used to model androgen effects on prostate cell gene expression. In our stromal cell model, AR alone is remarkably suppressive of gene expression, yet this effect does not alter their superficial cell morphology nor growth behavior. We believe this effect involves interaction of AR with the prostate stromal cell genome since it can often be reversed or augmented when ligand is provided. Through the use of gene profiling technology, we have provided a preliminary list of genes that are affected by liganded AR function and several of these same genes are also affected by DHT treatment of other types of prostate stromal cells that overexpress exogenous AR or primary prostate stromal cells that simply express low levels of endogenous AR. Many of the androgen affected genes are associated with signaling pathways involved in stromal-epithelial cell cross-talk in the prostate or with cancer pathways. This latter category of genes affected by androgens was consistent with our findings that conditioned medium from androgen-stimulated WPMY-AR cells more support prostate cancer cell growth than from androgen-stimulated control cells that lack AR. Collectively, the work represents a preliminary characterization that can be extended in the future to significantly enhance our understanding of androgen function in human prostate stromal cells.

## Materials and Methods

### Cells and Reagents

Benign immortalized human prostate stromal cells, WPMY-1 [20] were obtained from ATCC (Manassas, VA); PS30 cells [25] were kindly provided by Debra Schwinn (Duke University, NC); and primary human prostate stromal fibroblasts were grown from a non-cancerous region of a human prostate [33] as previously described. Human prostate cancer, LNCaP cells, were purchased from ATCC. PS30 and LNCaP cells were cultured in RPMI-1640 (Hyclone, Waltham, MA) supplemented with 10% heat-inactivated fetal bovine serum (FBS) (Hyclone), 1% penicillin/streptomycin, 1% glutamine and 1% sodium pyruvate (Invitrogen, Inc., Carlsbad, NC). WPMY cells were cultured in Dulbecco's Modified Eagle's Medium (Hyclone) supplemented 10% FBS, 1% penicillin/streptomycin, 1% glutamine and 1% sodium pyruvate. Charcoal/dextran-Stripped FBS (CS-FBS) was obtained from Hyclone. Dihydrotestosterone (DHT) was obtained from Sigma-Aldrich Chemical Co. (St. Louis, MO). Mouse monoclonal anti-human AR antibody (clone 441) and mouse monoclonal anti-human GAPDH (clone 6C5) was purchased from Santa Cruz Biosciences (Santa Cruz, CA). Secondary sheep anti-mouse HRP was purchased from GE Healthcare (Pittsburgh, PA). Blastocidin S HCl was purchased from Invitrogen (Carlsbad, CA).

### DNA Vectors and Cell Manipulation Procedures

An androgen reporter vector with a synthetic androgen-responsive promoter (ARE-Luc, Panomics, Inc., Fremont, CA) and pEGFP (Clontech, Mountain View, CA) were transfected into cells using Lipofectamine 2000 (Invitrogen). Replication-deficient lentivirus pLenti6-hAR was derived from inserting the human AR full length wildtype cDNA into the pLenti6.2 plasmid (Invitrogen). Conditioned medium containing infectious virus was obtained by transfection of 293FT HEK cells with pLenti6-hAR or pLenti6.2 (empty vector control) along with accessory lentiviral packaging plasmids VSV-G and delta 8.91. Medium from these transfected cells was collected 48 hrs after transfection and was filtered. Stable cells were derived after incubation with viral conditioned medium for 48 hrs followed by selection in fresh medium containing in Blasticidin S (1 µg/ml, Invitrogen) and were pooled and designated WPMY-Vec (pLenti6.2 empty vector) or WPMY-AR (pLenti6-hAR).

### Gene Expression Profiling Using Gene Chip Microarrays

WPMY-Vec or WPMY-AR cells were trypsinized then plated at 1×10^6^ cells per 60 mm dish in DMEM with 10% FBS. After overnight attachment, medium was removed, plates were rinsed with PBS and fresh medium with 10% CS-FBS, with or without 10 nM DHT was added and cells were maintained for 72 hrs. Cells were washed with PBS then lysed and RNA was purified with the RNEasy Plus micro kit (Qiagen Inc., Valencia, CA) as directed by the manufacturer. Individual RNAs were analyzed for RNA quality by Bioanalyzer Chips (Agilent Technologies, Santa Clara, CA) and only RNAs with a RIN of 9.0 or higher we used for subsequent gene expression profiling. RNA labeling and hybridization were performed by the Ordway Research Institute microarray core facility according to the Affymetrix microarray analysis protocols. Briefly, single-standed cDNA was generated from amplified cRNA with the WT cDNA Synthesis Kit (Affymetrix, Santa Clara, CA) and then fragmented a labeled with the WT Terminal Labeling Kit (Affymetrix) s. Samples were hybridized with Affymetrix Human ST 1.0 Gene Chips (Affymetrix) and scanned on the Affymetrix Gene Chip Scanner 3000 in the core facility and were collected into CEL files for further analysis. Resulting signal analysis was performed with GeneSpring GX 11.0.2 (Agilent Technologies) software. Expressions of genes under different conditions was filtered by statistical significance (students T-test, p>0.05) by GeneSpring program and comparisons between treatment groups fold induction cut-offs of 1.5 or 2.0 fold or higher between sample groups.

### Quantitative Real-Time RT-PCR

Cells (biological duplicate specimens) were lysed and total RNAs were extracted using the RNeasy mini-kit (Qiagen, Inc.). RNA concentrations were estimated by absorbance at 260 nm. First strand cDNA synthesis was performed using the SuperScript™ III First-Strand Synthesis System for qRT-PCR (Invitrogen). Gene-specific primer sets used for real-time analysis are described in Table S1. Primer sets (0.5 µM) were mixed with cDNA template and RT^2^ SYBR Green Master Mix (SABiosciences, Inc., Frederick, MD), and qRT-PCR was performed using an ABI Prism 7900 HT sequence detector as previously described [34]. Relative mRNA expression levels were determined by comparison to the GAPDH internal control and plotted as ratio to GAPDH expression values.

### Luciferase Assay

Cells were seeded into 6 well plates at 2×10^5^ cells per well. After overnight attachment, cells were transfected with 2 µg pLenti6.2 or pLenti6-hAR with 1.5 µg ARE-Luc reporter vector and 0.5 µg pEGFP. Medium was changed after 4 hrs to DMEM with 10% CS-FBS with or without DHT as indicated. After 72 hrs, medium was removed, cells were lysed in 1% Triton X-100 buffer, and the lysates analyzed by on a Fluostar Optima fluorometer (for GFP fluorescence) (BMG Labtechnologies, Durham, NC) and on a 20/20n Luminometer (Turner Biosystems, Sunnyvale, CA) after incubation with firefly luciferase reagent (Promega, Inc., Madison, WI). GFP values were used to normalize luciferase values and data is presented as a ratio of luciferase to GFP levels.

### Western Blot Analysis

Sub-confluent monolayers of cells were lysed and their protein contents measured as was previously described [35]. SDS-PAGE loading dye was added to aliquots containing equal protein amounts from each cell line, boiled, and loaded onto an SDS-PAGE gel for electrophoresis. The gel was electro-transferred to a nitrocellulose membrane, blocked in 5% milk, and probed with anti-AR or anti-GAPDH antibodies overnight. The membrane was then washed, and probed with sheep anti-mouse conjugated HRP (GE Healthcare, UK). After incubation, the membrane was washed, treated with ECL reagent (SuperSignal West Pico Chemiluminescent Substrate, Thermo Scientific, Rockford, IL) and exposed to x-ray film.

### Conditioned Medium Preparation and LNCaP Proliferation Assay

WPMY1-Vector or WPMY1-AR cells were plated at 3×10^6^ cells in a 100 mm culture dish in RPMI with 10% CS-FBS supplemented with 10 nM DHT and grown for 72 hrs. Medium was then filtered through a 0.22 µm filter and used immediately or frozen. LNCaP cells were plated in 96 well plates (5000 cells/well) in replicates of 6 wells per assay condition or incubation day. After attachment, medium was removed and wells were treated with a 1∶1 mixture of RPMI, 10% CS-FBS, 10 nM DHT with 72 hr conditioned medium from WPMY1-Vector or WPMY1-AR cells. Medium was changed every three days. WST-1 reagent (Dojindo Laboratories, Kamimashiki, Japan) was added to individual wells at 3-day intervals for 9 days and the plate was then read after 90 min at 450 nm (SpectraMax M2 plate reader, Molecular Diagnostics, Sunnyvale, CA). Values for six samples at each point were averaged and data was graphed as absorbance vs time.

### Statistical Analysis

Comparative quantitative RT-PCR outcomes from DHT-untreated/-treated cells were based on 2 measurements each from 2 biological replicate samples and they were statistically analyzed using a two-tailed students T test. Differences in the growth curves of LNCaP cells grown with different conditioned stromal cell medium were analyzed by two-way Anova and curve slopes were compared using multiple linear regression analysis. P Values of ≤0.05 were considered significant.

## Supporting Information

Table S1 **Primers used in qPCR reactions**.
(DOC)

Table S2 **Genes up-regulated by 2-fold or greater in WPMY-AR cells by DHT.** * Indicates genes that were previously described to be androgen regulated.
(DOC)

Table S3 **Genes down-regulated by 2-fold or greater in WPMY-AR cells by DHT.** * Indicates genes that were previously described to be androgen regulated.
(DOC)

Figure S1 **AR expression in primary human prostate stromal cells (PrSC).** Primary cell cultures were incubated for 24 h with vehicle (ethanol, a) or 10 nM DHT (b) before immunostaining. Cells were fixed in 4% p-formaldehyde and stained with a polyclonal antibody against AR (Santa Cruz Biotechnology). Immunoreaction was visualized using a secondary antibody HRP-conjugated. AR nuclear translocation was evident in presence of DHT (b-c, high magnification picture). Images a and b: x300. Image c: x600.
(TIF)

## References

[1] Imperato-McGinley J, Zhu YS (2002). Androgens and male physiology the syndrome of 5alpha reductase-2 deficiency.. Mol Cell Endocrinol.

[2] Sultan C, Lumbroso S, Poujol N, Belon C, Boudon C (1993). Mutations of androgen receptor gene in androgen insensitivity syndromes.. J Steroid Biochem Mol Biol.

[3] Thompson IM, Tangen CM, Goodman PJ, Lucia MS, Klein EA (2009). Chemoprevention of prostate cancer.. J Urol.

[4] Miyamoto H, Messing EM, Chang C (2004). Androgen deprivation therapy for prostate cancer: current status and future prospects.. Prostate.

[5] Dehm SM, Tindall DJ (2006). Molecular regulation of androgen action in prostate cancer.. J Cell Biochem.

[6] Xu LL, Su YP, Labiche R, Segawa T, Shanmugam N (2001). Quantitative expression profile of androgen-regulated genes in prostate cancer cells and identification of prostate-specific genes.. Int J Cancer.

[7] Velasco AM, Gillis KA, Li Y, Brown EL, Sadler TM (2004). Identification and validation of novel androgen-regulated genes in prostate cancer.. Endocrinol.

[8] Jariwala U, Prescott J, Jia L, Barski A, Pregizer S (2007). Identification of novel androgen receptor target genes in prostate cancer.. Mol Cancer.

[9] Ngan S, Stronach EA, Photiou A, Waxman J, Ali S (2009). Microarray coupled to quantitative RT-PCR analysis of androgen-regulated genes in human LNCaP prostate cancer cells.. Oncogene.

[10] Liu AY, Corey E, Vessella RL, Lange PH, True LD (1997). Identification of differentially expressed prostate genes: increased expression of transcription factor ETS-2 in prostate cancer.. Prostate.

[11] Petrovics G, Liu A, Shaheduzzaman S, Furusato B, Sun C (2005). Frequent overexpression of ETS-related gene-1 (ERG1) in prostate cancer transcriptome.. Oncogene.

[12] Rostad K, Mannelqvist M, Halvorsen OJ, Oyan AM, Bo TH (2007). ERG upregulation and related ETS transcription factors in prostate cancer.. Int J Oncol.

[13] Hu P, Greenwood CM, Beyene J (2007). Integrative analysis of gene expression data including an assessment of pathway enrichment for predicting prostate cancer.. Cancer Inform.

[14] Gorlov IP, Byun J, Gorlova OY, Aparicio AM, Efstathiou E (2009). Candidate pathways and genes for prostate cancer: a meta-analysis of gene expression data.. BMC Med Genomics.

[15] Gleave M, Hsieh JT, Gao CA, von Eschenbach AC, Chung LW (1991). Acceleration of human prostate cancer growth in vivo by factors produced by prostate and bone fibroblasts.. Cancer Res.

[16] Cunha GR, Hayward SW, Wang YZ (2002). Role of stroma in carcinogenesis of the prostate.. Differentiation.

[17] Cunha GR, Hayward SW, Wang YZ, Ricke WA (2003). Role of the stromal microenvironment in carcinogenesis of the prostate.. Int J Cancer.

[18] Cunha GR, Ricke W, Thomson A, Marker PC, Risbridger G (2004). Hormonal, cellular, and molecular regulation of normal and neoplastic prostatic development.. J Steroid Biochem Mol Biol.

[19] Chung LW, Baseman A, Assikis V, Zhau HE (2005). Molecular insights into prostate cancer progression: the missing link of tumor microenvironment.. J Urol.

[20] Webber MM, Trakul N, Thraves PS, Bello-DeOcampo D, Chu WW (1999). A human prostatic stromal myofibroblast cell line WPMY-1: a model for stromal-epithelial interactions in prostatic neoplasia.. Carcinogenesis.

[21] Draghici S, Khatri P, Tarca AL, Amin K, Done A (2007). A systems biology approach for pathway level analysis.. Genome Res.

[22] Sar M, Lubahn DB, French FS, Wilson EM (1990). Immunohistochemical localization of the androgen receptor in rat and human tissues.. Endocrinol.

[23] Iwamura M, Abrahamsson PA, Benning CM, Cockett AT, di Sant'Agnese PA (1994). Androgen receptor immunostaining and its tissue distribution in formalin-fixed, paraffin-embedded sections after microwave treatment.. J Histochem Cytochem.

[24] Mohler JL, Chen Y, Hamil K, Hall SH, Cidlowski JA (1996). Androgen and glucocorticoid receptors in the stroma and epithelium of prostatic hyperplasia and carcinoma.. Clin Cancer Res.

[25] Price DT, Rudner X, Michelotti GA, Schwinn DA (2000). Immortalization of a human prostate stromal cell line using a recombinant retroviral approach.. J Urol.

[26] Snoek R, Cheng H, Margiotti K, Wafa LA, Wong CA (2009). In vivo knockdown of the androgen receptor results in growth inhibition and regression of well-established, castration-resistant prostate tumors.. Clin Cancer Res.

[27] Heisler LE, Evangelou A, Lew AM, Trachtenberg J, Elsholtz HP (1997). Androgen-dependent cell cycle arrest and apoptotic death in PC-3 prostatic cell cultures expressing a full-length human androgen receptor.. Mol Cell Endocrinol.

[28] Jiang M, Ma Y, Chen C, Fu X, Yang S (2009). Androgen-responsive gene database: integrated knowledge on androgen-responsive genes.. Mol Endocrinol.

[29] Shigemura K, Isotani S, Wang R, Fujisawa M, Gotoh A (2009). Soluble factors derived from stroma activated androgen receptor phosphorylation in human prostate LNCaP cells: roles of ERK/MAP kinase.. Prostate.

[30] Basanta D, Strand DW, Lukner RB, Franco OE, Cliffel DE (2009). The role of transforming growth factor-beta-mediated tumor-stroma interactions in prostate cancer progression: an integrative approach.. Cancer Res.

[31] Li X, Placencio V, Iturregui JM, Uwamariya C, Sharif-Afshar AR (2008). Prostate tumor progression is mediated by a paracrine TGF-beta/Wnt3a signaling axis.. Oncogene.

[32] Unni E, Sun S, Nan B, McPhaul MJ, Cheskis B (2004). Changes in androgen receptor nongenotropic signaling correlate with transition of LNCaP cells to androgen independence.. Cancer Res.

[33] Cano P, Godoy A, Escamilla R, Dhir R, Onate SA (2007). Stromal-epithelial cell interactions and androgen receptor-coregulator recruitment is altered in the tissue microenvironment of prostate cancer.. Cancer Res.

[34] Mechlin CW, Tanner MJ, Chen M, Buttyan R, Levin RM (2010). Gli2 expression and human bladder transitional carcinoma cell invasiveness.. J Urol.

[35] Chen M, Tanner M, Levine AC, Levina E, Ohouo P (2009). Androgenic regulation of hedgehog signaling pathway components in prostate cancer cells.. Cell Cycle.

