# Supplementary material for: Effects of Androgen Receptor and Androgen on Gene Expression in Prostate Stromal Fibroblasts and Paracrine Signaling to Prostate Cancer Cells
Source: PLoS One. 2011 Jan 18;6(1):e16027. doi: 10.1371/journal.pone.0016027 (PMC3022749; doi:10.1371/journal.pone.0016027)
Supplement: Table S1 — Primers used in qPCR reactions. (DOC) [file pone.0016027.s001.doc]

**Table S1. Primers Sets Used for Real-Time PCR**

| **Primer Name** | **Forward (5’-3’)** | **Reverse (5’-3’)** |
| --- | --- | --- |
| APOD | CAGCGTCCATTCTCAAAGGT | CTCCGGTGCAGGAGAATTT |
| AQP3 | AGATGCTCCACATCCGCTAC | GGTTGATGGTGAGGAAACCA |
| AR | CCTGGCTTCCGCAACTTACAC | GGACTTGTGCATGCGGTACTCA |
| BMP4 | GCATTCGGTTACCAGGAATC | TGAGCCTTTCCAGCAAGTTT |
| CCRL1 | CCTCTCTGCCGACTACAACA | GCAAATCTCTGACATCTTCTTTG |
| FGF5 | ATTTGCTGTGTCTCAGGGGA | GAAAACGCTCCCTGAACTTG |
| FKBP5 | CTGAAGGGTTAGCGGAGCA | CTGTGGGGCTTTCTTCATTG |
| FST | CTTTGCCTCCTGCTGCTG | ACTCCTCCTTGCTCAGTTCG |
| GAPDH | CCATCACCATCTTCCAGGAGCG | AGAGATGATGACCCTTTTGGC |
| IGF1 | GCTCTTCAGTTCGTGTGTGG | CGCAATACATCTCCAGCCTC |
| IL7R | CTCATGCTATAGCCAGTTGGA | TCAGGCACTTTACCTCCACG |
| KLK2 | GCTGCCCATTGCCTAAAGAAG | TGGGAAGCTGTGGCTGACA |
| KLK3 (PSA) | TATTGTAGTAAACTTGGAACCTTG | TTACACCATTTAAGAAACACTCTG |
| LCN8 | CGGGGCAGGAACTTTCGCGT | CTCGGCACACCGCCCAGG |
| LRRFIP1 | GGTAGTCGTGGAAGCCTGAG | GAGAAGGAGGTGTCTCCGCT |
| p21 | CCCAAGCTCTACCTTCCCAC | ACAAGACAGTGACAGGTCCAC |
| p27 | ACAGAAGAAAATGTTTCAGACGGT | CTTCTGAGGCCAGGCTTCTT |
| RERG | GCTAAAAGTGCGGAGGTCAA | GTTGCTTGGTGTCGGTAGGT |
| Sept9 | CGCCGCTGCTAAATATATCC | CTCGGAGTAGGGGAGTCTGG |
| SFRP5 | CCAGTGTGAGATGGAGCACA | GGCTTGAGCAGCTTCTTCTT |
| Wnt16 | CACGGGCAAAGAAAACAAAG | GCATGTTTTCACAGCACAGG |
