# Supplementary material for: Effects of Androgen Receptor and Androgen on Gene Expression in Prostate Stromal Fibroblasts and Paracrine Signaling to Prostate Cancer Cells
Source: PLoS One. 2011 Jan 18;6(1):e16027. doi: 10.1371/journal.pone.0016027 (PMC3022749; doi:10.1371/journal.pone.0016027)
Supplement: Table S2 — Genes up-regulated by 2-fold or greater in WPMY-AR cells by DHT. * Indicates genes that were previously described to be androgen regulated. (DOC) [file pone.0016027.s002.doc]

| **Genbank Accession** | **Gene Symbol** | **Gene Name** | **Upregulated**  **(Fold)** |
| --- | --- | --- | --- |
| AY154470 | RASSF2 | Ras association (RalGDS/AF-6) domain family member 2 | 9.4 |
| AK095363 | AQP3 | aquaporin 3 (Gill blood group) | 8.8 |
| NM_020721.1 | RP13-347D8.3 | KIAA1210 protein | 7.7 |
| BC063634 | MAMDC2 | MAM domain containing 2 | 6.6 |
| U70867 | SLCO2A1 | solute carrier organic anion transporter family, member 2A1 | 5.5 |
| AF040709 | CACNA2D2 | calcium channel, voltage-dependent, alpha 2/delta subunit 2 | 5.5 |
| AF169963 | WNT16 | wingless-type MMTV integration site family, member 16 | 5.2 |
| BC046629 | KCNG1 | potassium voltage-gated channel, subfamily G, member 1 | 5.1 |
| BC125069 | ZNF114 | zinc finger protein 114 | 5.0 |
| BC066334 | C13orf15 | chromosome 13 open reading frame 15 | 4.9 |
| AF368463 | CPM | carboxypeptidase M | 4.9 |
| BC042605 | FKBP5 | FK506 binding protein 5 | 4.9 |
| AF153416 | EPB41L4B | erythrocyte membrane protein band 4.1 like 4B | 4.8 |
| AK124901 | SLC16A12 | solute carrier family 16, member 12 (monocarboxylic acid transporter 12) | 4.8 |
| BX538010 | NRCAM | neuronal cell adhesion molecule | 4.8 |
| AF069506 | RASD1 | RAS, dexamethasone-induced 1 | 4.7 |
| BC022250 | C5orf23 | chromosome 5 open reading frame 23 | 4.6 |
| BC007997 | RERG | RAS-like, estrogen-regulated, growth inhibitor | 4.5 |
| BC007060 | AMY2 | amylase, alpha 2A (pancreatic)(pancreatic) | 4.4 |
| AF079529 | PDE8B | phosphodiesterase 8B | 4.4 |
| BC071561 | LRIG1 | leucine-rich repeats and immunoglobulin-like domains 1 | 4.4 |
| AF073299 | SLC9A2 | solute carrier family 9 (sodium/hydrogen exchanger), member 2 | 4.4 |
| BC012064 | PCSK5 | proprotein convertase subtilisin/kexin type 5 | 4.2 |
| BC050350 | TIPARP | TCDD-inducible poly(ADP-ribose) polymerase | 4.2 |
| AF538954 | GPR64 | G protein-coupled receptor 64 | 4.1 |
| AY358925 | LYVE1 | lymphatic vessel endothelial hyaluronan receptor 1 | 4.1 |
| AB037669 | SLC7A8 | solute carrier family 7 (cationic amino acid transporter, y+ system), member 8 | 4.1 |
| BC047295 | GPM6B | glycoprotein M6B | 4.0 |
| AK289988 | SMOC1 | SPARC related modular calcium binding 1 | 3.8 |
| AK290870 | NPR3 | natriuretic peptide receptor C/guanylate cyclase C (atrionatriuretic peptide receptor C) | 3.7 |
| AF232905 | C1QTNF1 | C1q and tumor necrosis factor related protein 1 | 3.7 |
| BC063129 | AMY1A | amylase, alpha 1A (salivary) | 3.6 |
| AF081195 | RASGRP1 | RAS guanyl releasing protein 1 | 3.5 |
| AF117758 | SFRP5 | secreted frizzled-related protein 5 | 3.4 |
| BC015875 | SEPP1 | selenoprotein P, plasma, 1 | 3.4 |
| BC111690 | SCUBE2 | signal peptide, CUB domain, EGF-like 2 | 3.3 |
| BC017583 | SCRG1 | scrapie responsive protein 1 | 3.2 |
| BC028280 | MT1M | metallothionein 1M | 3.2 |
| BC125017 | KIAA1772 | KIAA1772 Protein | 3.0 |
| AK001623 | ETNK2 | ethanolamine kinase 2 | 2.9 |
| AF096870 | TRIM16|CDRT1 | tripartite motif-containing 16 | 2.8 |
| AF127138 | LPAR3 | lysophosphatidic acid receptor 3 | 2.7 |
| BC036662 | SLC46A3 | solute carrier family 46, member 3 | 2.7 |
| AY358460 | ACPL2 | acid phosphatase-like 2 | 2.7 |
| AF423422 | STEAP4 | STEAP family member 4 | 2.6 |
| AY358713 | CCDC126 | coiled-coil domain containing 126 | 2.6 |
| AK074518 | OSR2 | odd-skipped related 2 (Drosophila) | 2.5 |
| BC032558 | DLX2 | distal-less homeobox 2 | 2.5 |
| L07615 | NPY1R | neuropeptide Y receptor Y1 | 2.4 |
| BC018986 | HPGD | hydroxyprostaglandin dehydrogenase 15-(NAD) | 2.4 |
| AY147881 | TMEM64 | transmembrane protein 64 | 2.3 |
| BC117309 | KIAA1324L | KIAA1324-like | 2.3 |
| AY367054 | FGD4 | FYVE, RhoGEF and PH domain containing 4 | 2.1 |
| BC114918 | MERTK | c-mer proto-oncogene tyrosine kinase | 2.0 |

**Table S2.** Genes up-regulated by 2-fold or greater in WPMY-AR cells by DHT*

*** Shaded boxes indicate genes previously found to be androgen-regulated**
