# Supplementary material for: Effects of Androgen Receptor and Androgen on Gene Expression in Prostate Stromal Fibroblasts and Paracrine Signaling to Prostate Cancer Cells
Source: PLoS One. 2011 Jan 18;6(1):e16027. doi: 10.1371/journal.pone.0016027 (PMC3022749; doi:10.1371/journal.pone.0016027)
Supplement: Table S3 — Genes down-regulated by 2-fold or greater in WPMY-AR cells by DHT. * Indicates genes that were previously described to be androgen regulated. (DOC) [file pone.0016027.s003.doc]

**Supplemental Table 3.** Genes down-regulated by 2-fold or greater in WPMY-AR cells by DHT*

| **Genbank Accession** | **Gene Symbol** | **Gene Name** | **Downregulated**  **(Fold)** |
| --- | --- | --- | --- |
| BC005926 | EVI2B | ecotropic viral integration site 2B | -6.6 |
| DQ346664 | RGS4 | regulator of G-protein signaling 4 | -5.3 |
| AF329499 | RAB27B | RAB27B protein | -5.2 |
| BC067540 | IL7R | interleukin 7 receptor | -5.0 |
| BC065937 | NT5E | 5'-nucleotidase, ecto (CD73) | -4.5 |
| BC017667 | NAV3 | neuron navigator 3 | -4.5 |
| AF062075 | LPXN | leupaxin | -4.4 |
| BC131502 | FGF5 | fibroblast growth factor 5 | -4.2 |
| AL834247 | MYPN | myopalladin | -4.2 |
| AY057053 | ASB5 | ankyrin repeat and SOCS box-containing 5 | -4.2 |
| AY266351 | IL18 | interleukin 18 (interferon-gamma-inducing factor) | -4.1 |
| Top of Form  NM_002203.3 | ITGA2 | integrin, alpha 2 (CD49B, alpha 2 subunit of VLA-2 receptor) | -4.1 |
| BX537386 | F2RL2 | coagulation factor II (thrombin) receptor-like 2 | -4.0 |
| AK125647 | MALL | mal, T-cell differentiation protein-like | -4.0 |
| BC004107 | FST | follistatin | -4.0 |
| BC052289 | CPA4 | carboxypeptidase A4 | -4.0 |
| U10550 | GEM | GTP binding protein overexpressed in skeletal muscle | -3.9 |
| BC036515 | ADAMTS1 | ADAM metallopeptidase with thrombospondin type 1 motif, 1 | -3.8 |
| AK122672 | GPRC5A | G protein-coupled receptor, family C, group 5, member A | -3.7 |
| AF223574 | PLK2 | polo-like kinase 2 (Drosophila) | -3.7 |
| AF023476 | ADAM12 | ADAM metallopeptidase domain 12 (meltrin alpha) | -3.7 |
| BC048284 | CDA | cytidine deaminase | -3.7 |
| BC009716 | CCL2 | chemokine (C-C motif) ligand 2 | -3.6 |
| BC150190 | ITGA3 | integrin, alpha 3 (antigen CD49C, alpha 3 subunit of VLA-3 receptor) | -3.6 |
| BC095403 | PLAT | plasminogen activator, tissue | -3.6 |
| BC063685 | VEGFC | vascular endothelial growth factor C | -3.6 |
| BC014439 | SPHK1 | sphingosine kinase 1 | -3.5 |
| BC015557 | CD68|EIF4A1 | CD68 molecule | eukaryotic translation initiation factor 4A, isoform 1 | -3.5 |
| J03634 | INHBA | inhibin, beta A | -3.5 |
| AY026461 | CDCP1 | CUB domain containing protein 1 | -3.5 |
| BC032495 | GDF5 | growth differentiation factor 5 | -3.3 |
| AF004711 | KCNK2 | potassium channel, subfamily K, member 2 | -3.2 |
| AY424283 | PAQR5 | progestin and adipoQ receptor family member V | -3.2 |
| BC036666 | AK5 | adenylate kinase 5 | -3.2 |
| U03688 | CYP1B1 | cytochrome P450, family 1, subfamily B, polypeptide 1 | -3.2 |
| BC016648 | FOSL1 | FOS-like antigen 1 | -3.1 |
| AB071179 | NEDD4L | neural precursor cell expressed, developmentally down-regulated 4-like | -3.1 |
| AF344424 | PDCD1LG2 | programmed cell death 1 ligand 2 | -3.1 |
| Y13786 | ADAM19 | ADAM metallopeptidase domain 19 (meltrin beta) | -3.0 |
| BC008832 | HMGA1 | high mobility group AT-hook 1 | -3.0 |
| D31784 | CDH6 | cadherin 6, type 2, K-cadherin (fetal kidney) | -2.9 |
| AY358803 | UNQ1940 | HWKM1940 | -2.9 |
| AY232290 | GREM1 | gremlin 1, cysteine knot superfamily, homolog (Xenopus laevis) | -2.9 |
| AK131447 | SPOCD1 | SPOC domain containing 1 | -2.9 |
| BC020546 | BMP4 | bone morphogenetic protein 4 | -2.9 |
| AF089841 | FLNC | filamin C, gamma (actin binding protein 280) | -2.8 |
| AJ001348 | LY6K | lymphocyte antigen 6 complex, locus K | -2.8 |
| BC053895 | IRS1 | insulin receptor substrate 1 | -2.8 |
| BC005080 | IER3 | immediate early response 3 | -2.8 |
| BC026314 | SHC3 | SHC (Src homology 2 domain containing) transforming protein 3 | -2.8 |
| BC098448 | Mar4 | membrane-associated ring finger (C3HC4) 4 | -2.8 |
| L03427 | BNC1 | basonuclin 1 | -2.8 |
| AB051479 | VEPH1 | ventricular zone expressed PH domain homolog 1 (zebrafish) | -2.8 |
| BC050743 | SMAD3 | SMAD family member 3 | -2.8 |
| BC110820 | PHLDA1 | pleckstrin homology-like domain, family A, member 1 | -2.8 |
| BC032697 | FGF1 | fibroblast growth factor 1 (acidic) | -2.8 |
| BC022322 | GNPDA1 | glucosamine-6-phosphate deaminase 1 | -2.7 |
| BC101758 | APCDD1L | adenomatosis polyposis coli down-regulated 1-like | -2.7 |
| BC070040 | SLC6A15 | solute carrier family 6, member 15 | -2.7 |
| AF269162 | C21orf7 | chromosome 21 open reading frame 7 | -2.6 |
| M76125 | AXL | Human Tyrosine Receptor (axl) | -2.6 |
| **Genbank Accession** | **Gene Symbol** | **Gene Name** | **Fold Regulation** |
| BC030205 | TNFAIP6 | tumor necrosis factor, alpha-induced protein 6 | -2.6 |
| BC110918 | INPP4B | inositol polyphosphate-4-phosphatase, type II, 105kDa | -2.6 |
| U77085 | EMP1 | epithelial membrane protein 1 | -2.6 |
| BC125200 | SLFN5 | schlafen family member 5 | -2.5 |
| BC033803 | LSAMP | limbic system-associated membrane protein | -2.5 |
| BC041070 | KRT34 | keratin 34 | -2.4 |
| AF316032 | MPP4 | membrane protein, palmitoylated 4 (MAGUK p55 subfamily member 4) | -2.4 |
| AF438313 | TMEM158 | transmembrane protein 158 | -2.4 |
| BC013577 | CLDN11 | claudin 11 (oligodendrocyte transmembrane protein) | -2.3 |
| BC022988 | C6orf65 | chromosome 6 open reading frame 65 | -2.3 |
| BC000594 | LOXL2|ENTPD4 | lysyl oxidase-like 2 | -2.3 |
| BC017276 | VCAM1 | vascular cell adhesion molecule 1 | -2.3 |
| BC001109 | CRABP2 | cellular retinoic acid binding protein 2 | -2.3 |
| BC002472 | LPCAT2 | lysophosphatidylcholine acyltransferase 2 | -2.3 |
| BC009356 | CDC42EP1 | CDC42 effector protein (Rho GTPase binding) 1 | -2.2 |
| BC001653 | MLPH | melanophilin | -2.2 |
| AY358628 | LYPD1 | LY6/PLAUR domain containing 1 | -2.2 |
| AK292655 | SNTB1 | syntrophin, beta 1 (dystrophin-associated protein A1, 59kDa, basic component 1) | -2.2 |
| AF015257 | GPER | G protein-coupled estrogen receptor 1 | -2.2 |
| BC006428 | CXXC5 | CXXC finger 5 | -2.1 |
| AK092355 | ZBTB38 | zinc finger and BTB domain containing 38 | -2.1 |
| AY358306 | GPR125 | G protein-coupled receptor 125 | -2.0 |

*Shaded boxes indicate genes previously described to be androgen-regulated
