# Supplementary figures and images for: Effects of Androgen Receptor and Androgen on Gene Expression in Prostate Stromal Fibroblasts and Paracrine Signaling to Prostate Cancer Cells
Source: PLoS One. 2011 Jan 18;6(1):e16027. doi: 10.1371/journal.pone.0016027 (PMC3022749; doi:10.1371/journal.pone.0016027)

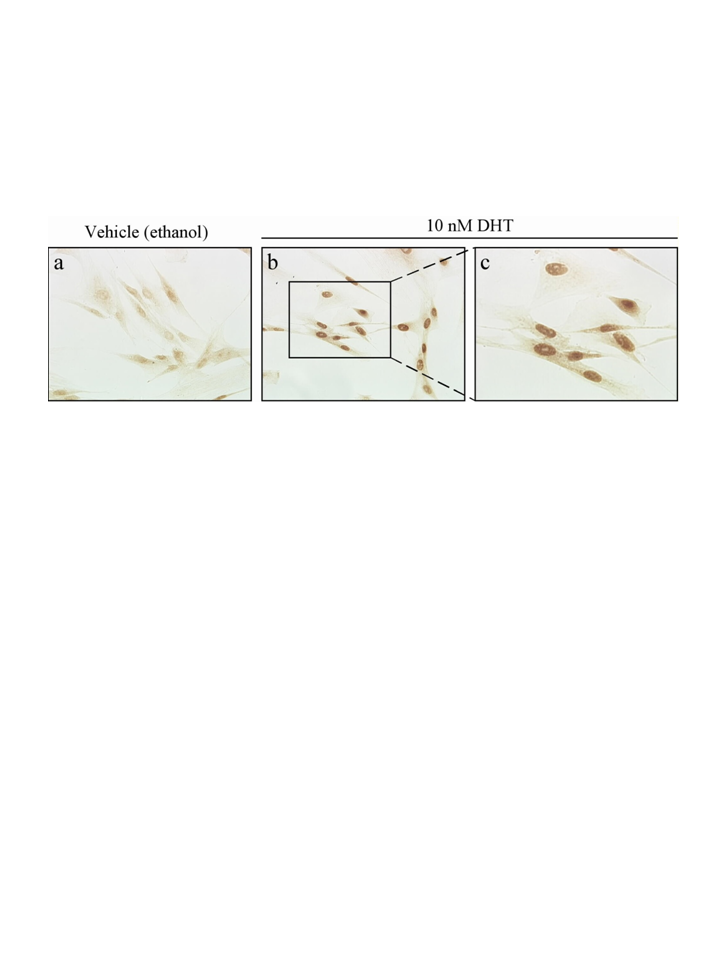

Supplement: Figure S1 — AR expression in primary human prostate stromal cells (PrSC). Primary cell cultures were incubated for 24 h with vehicle (ethanol, a) or 10 nM DHT (b) before immunostaining. Cells were fixed in 4% p-formaldehyde and stained with a polyclonal antibody against AR (Santa Cruz Biotechnology). Immunoreaction was visualized using a secondary antibody HRP-conjugated. AR nuclear translocation was evident in presence of DHT (b-c, high magnification picture). Images a and b: x300. Image c: x600. (TIF) [file pone.0016027.s004.tif]
